# Lipidome of Atherosclerotic Plaques from Hypercholesterolemic Rabbits

**DOI:** 10.3390/ijms151223283

**Published:** 2014-12-15

**Authors:** Lazar A. Bojic, David G. McLaren, Vinit Shah, Stephen F. Previs, Douglas G. Johns, Jose M. Castro-Perez

**Affiliations:** Merck Research Laboratories, 2000 Galloping Hill Road, Kenilworth, NJ 07033, USA; E-Mails: lazar.bojic@merck.com (L.A.B.); david_mclaren@merck.com (D.G.M.); vinit_shah2@merck.com (V.S.); stephen_previs@merck.com (S.F.P.); douglas_johns@merck.com (D.G.J.)

**Keywords:** lipidomics, atherosclerosis, plaque, lipids, mass spectrometry

## Abstract

The cellular, macromolecular and neutral lipid composition of the atherosclerotic plaque has been extensively characterized. However, a comprehensive lipidomic analysis of the major lipid classes within atherosclerotic lesions has not been reported. The objective of this study was to produce a detailed framework of the lipids that comprise the atherosclerotic lesion of a widely used pre-clinical model of plaque progression. Male New Zealand White rabbits were administered regular chow supplemented with 0.5% cholesterol (HC) for 12 weeks to induce hypercholesterolemia and atherosclerosis. Our lipidomic analyses of plaques isolated from rabbits fed the HC diet, using ultra-performance liquid chromatography (UPLC) and high-resolution mass spectrometry, detected most of the major lipid classes including: Cholesteryl esters, triacylglycerols, phosphatidylcholines, sphingomyelins, diacylglycerols, fatty acids, phosphatidylserines, lysophosphatidylcholines, ceramides, phosphatidylglycerols, phosphatidylinositols and phosphatidylethanolamines. Given that cholesteryl esters, triacylglycerols and phosphatidylcholines comprise greater than 75% of total plasma lipids, we directed particular attention towards the qualitative and quantitative assessment of the fatty acid composition of these lipids. We additionally found that sphingomyelins were relatively abundant lipid class within lesions, and compared the abundance of sphingomyelins to their precursor phosphatidylcholines. The studies presented here are the first approach to a comprehensive characterization of the atherosclerotic plaque lipidome.

## 1. Introduction

For the past century, cardiovascular disease (CVD) has been the leading cause of death in the industrialized world and is projected to soon achieve this status worldwide [1,2]. At the core of most cardiovascular events is atherosclerosis, a chronic inflammatory condition of the macrovasculature initiated by the subendothelial retention of apolipoprotein B (apoB)-containing lipoproteins [3]. The trapping of lipoproteins by proteoglycans within the arterial intima increases the propensity for various lipoprotein modifications (oxidation, hydrolysis, aggregation) which trigger maladaptive immune responses that potentiate lesion development [3]. Eventually, atherosclerotic lesions reach an unstable state that is prone to rupture, ultimately resulting in acute cardiovascular events [3,4].

Monogenic disorders in which plasma low-density lipoprotein cholesterol (LDL-C) levels are substantially elevated (such as familial hypercholesterolemia) result in significantly increased cardiovascular event rates, and a number of randomized controlled trials of LDL-C lowering interventions, namely statins, consistently demonstrate reductions in CVD risk [5]. As a result, this evidence places beyond a reasonable doubt that LDL-C is a causative biomarker of atherogenesis. Given this breadth of existing data surrounding the link between plasma cholesterol and atherosclerotic cardiovascular disease [5], a logical assumption would be that cholesterol predominates as the lipid species within atheromatas. However, two recent studies have suggested that in a hypercholesterolemic rabbit model of atherosclerosis, triacylglycerols (TGs) actually represent a major constituent of atherosclerotic plaques [6,7]. Despite these studies, a detailed lipidomic analysis of rabbit plaque composition has not yet been reported. Such information may highlight novel therapeutic strategies to induce the regression of established atherosclerotic lesions, which current LDL-lowering therapies have only modestly achieved [8].

The goal of this study was to construct a detailed framework of the lipids that comprise atherosclerotic lesions that could help us to identify novel treatment strategies that promote lesion regression. Here we report a comprehensive lipidomic analysis of atherosclerotic plaques that develop as a consequence of hypercholesterolemia. We took advantage of ultra-performance liquid chromatography (UPLC) and high-resolution mass spectrometry to gain insight into lesion composition. We quantified relative amounts of lipid subclasses based on acyl chain length and degree of acyl chain saturation within major lipid fractions detected in aortic plaques.

## 2. Results

Male New Zealand White rabbits were administered either regular chow (RC) or regular chow supplemented with 0.5% cholesterol (HC) for 12 weeks to induce hypercholesterolemia and atherosclerosis. Rabbits fed the HC diet developed hypercholesterolemia compared to rabbits fed the RC diet (43.65 ± 3.67 *versus* 0.93 ± 0.15 mmol/L) without an elevation in plasma TGs (0.65 ± 0.06 *versus* 0.53 ± 0.07 mmol/L) These data are consistent with recently published studies from our laboratory [9].

In order to assess plaque lipid content, we took advantage of high resolution time of flight (ToF) mass spectrometry which can delineate analytes with a mass accuracy of less than 5 parts per million (Tables S1–S14). Thus, we were able to accurately measure the relative quantities of lipids within the atherosclerotic plaque. We isolated lipids from the full-length of the aorta beginning with the aortic arch through the descending aorta to the iliac bifurcation, and subjected the plaque to LC-MS/MS on ToF. Our lipidomic analyses detected most of the major lipid classes including: Cholesteryl esters (CEs), TGs, phosphatidylcholines (PCs), sphingomyelins (SMs), diacylglycerols (DGs), fatty acids (FAs), phosphatidylserines (PSs), lysophosphatidylcholines (LPCs), ceramides (Cers), phosphatidylglycerols (PGs), phosphatidylinositols (PIs) and phosphatidylethanolamines (PEs). Herein, we present a relative quantitation of the fatty acyl composition of these lipid fractions, with particular attention to CEs, TGs, and PCs, the three plasma lipid classes that account for greater than 75% of total plasma lipid [10]. We additionally focused on the lipidomics of plaque SMs, as we found this lipid class to be relatively abundant in lesions. The remaining lipidomic analyses of the lipid classes mentioned above have been cataloged in the supplemental material (Tables S5–S14).

**Figure 1:**
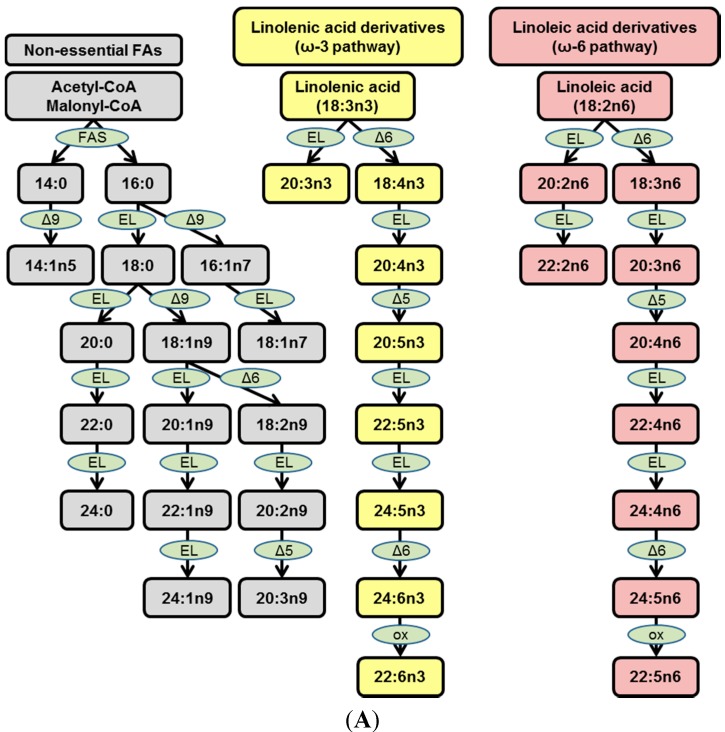

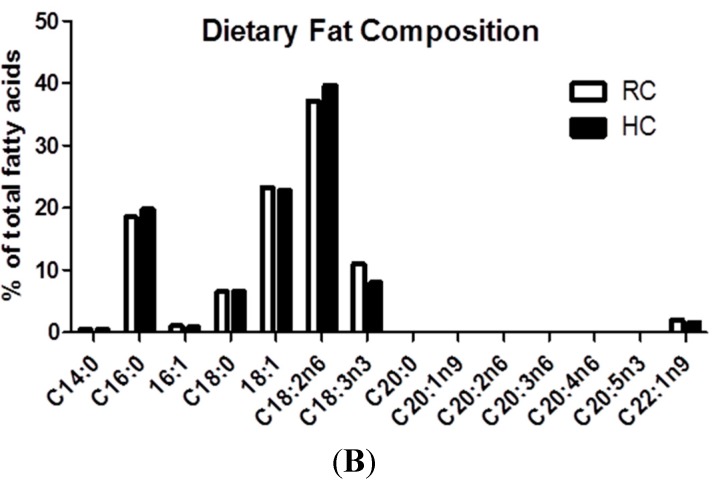
Fatty acid (FA) biosynthetic pathways and rabbit diet fatty acyl composition. (**A**) Schematic representation of three major FA biosynthetic pathways: Nonessential FAs (grey), ω-3 FAs (yellow), ω-6 FAs (lavender). Enzymes are depicted in light green; and (**B**) FA composition of rabbit diets. Regular chow (RC) and high cholesterol (HC).

Fatty acids identified in plasma lipids are often categorized as: (i) Nonessential FAs, namely saturated FAs (SFAs) and mono- or polyunsaturated FAs (MUFAs and PUFAs); (ii) Essential FAs that are metabolites from either the ω-3 pathway (linolenic acid derivatives) or the ω-6 pathway (linoleic acid derivatives). Nonessential FAs can originate from the diet or from *de novo* lipogenesis (DNL), whereas essential FAs can only originate from the metabolism of essential ω-3 or ω-6 FAs obtained from diet (Figure 1A and [9]).

**Figure 2:**
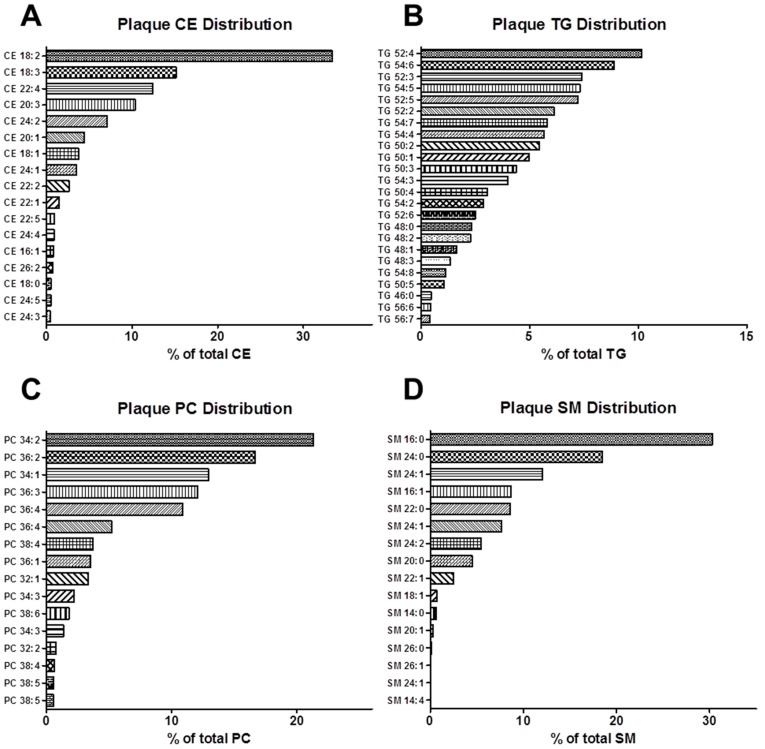
Fatty acyl composition of plaque lipids: Targeted analysis of lipids was conducted on full-length aortae isolated from rabbits fed the HC diet by UPLC/TOF-MS. (**A**) Plaque cholesteryl esters (CE); (**B**) Plaque triacylglycerols (TGs); (**C**) Plaque phosphatidylcholines (PCs); and (**D**) Plaque sphingomyelins (SMs). All data is presented as the percent of the given lipid species of the total lipid class.

We have recently shown that in cholesterol-fed rabbits, over 75% of the FAs within plasma CEs are SFAs and MUFAs, suggesting a strong dietary influence on the plasma lipidome [9]. Given the dietary composition of the diet in this study (Figure 1B), the CE 18:2 and 18:3 species that predominate as the CE species within rabbit plaque (Figure 2A, Table S1) are most likely CE 18:2n6 and CE 18:2n3, both of which are derived from diet. Together with CE 20:1, 18:1, 24:1 and 22:1, over 60% of lesion CEs can be attributed to dietary or nonessential FA contributions (Figure 2A, Table S1). Similar to plasma [9], ω-6 FAs are the next major constituents of plaque CEs, with CE 22:4, 20:3 and 22:2 accounting for another 25% of fatty acyl CEs within rabbit lesions (Figure 2A, Table S1).

Rabbits administered the HC diet had identical FA composition of plasma TGs compared to rabbits administered the RC diet, with nearly 80% of FAs within TGs being derived from nonessential FAs [9]. Here, we find that the most abundant TGs in plaque are comprised of FAs obtained from the diet, accounting for over 80% of TGs in plaque (Figure 2B, Table S2). Akin to our findings in the plasma, metabolites of the ω-3 and ω-6 pathways are likely only minor contributors of TG deposition to the plaque (Figure 2B, Table S2).

In our previous report, 55% of FAs in PCs came from nonessential FAs, 40% came from the ω-6 pathway and the remaining 5% came from the ω-3 pathway [9]. Interestingly, 91% of the FAs in plaque PCs stemmed from dietary nonessential FAs and ~8% stemmed from metabolites of the ω-6 pathway (Figure 2C, Table S3), suggesting that the dietary FAs in circulating PCs are highly prone to deposition within atherosclerotic plaque PCs.

**Figure 3:**
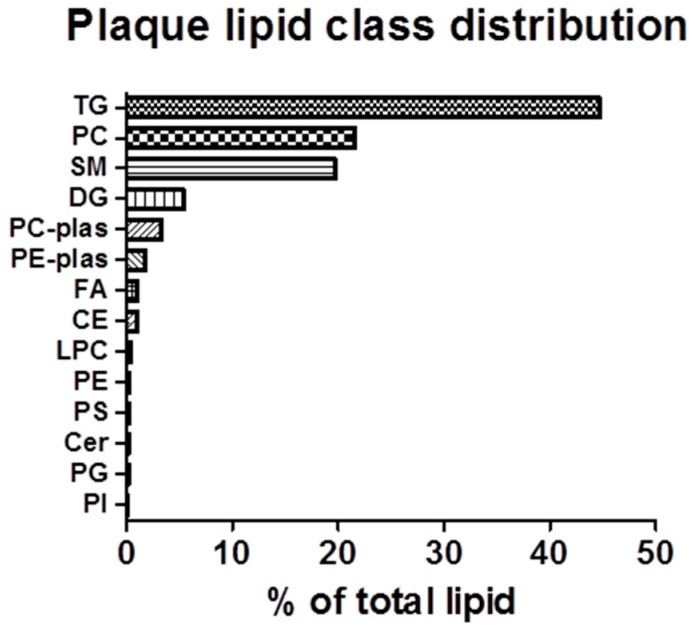
Summary of aortic lipid class distribution: Targeted analysis of lipids was conducted on full-length aortae isolated from rabbits fed the HC diet by UPLC/TOF-MS. Data is expressed as the percent of the lipid class of the total lipid content in the aorta, calculated as the total peak area of each lipid class, divided by the total peak area of all lipid classes. TG: Triacylglycerol; SM: Sphingomyelin; PC: Phosphatidylcholine; DG: Diacylglycerol; FA: Fatty acid; PE-plas: Phosphatidylthanolamine; PC-plas: Phosphatidyl choline; CE: Cholesteryl ester; Cer: Ceramide; PS: Phosphatidylserine; LPC: Lyso-phosphatidic acid; PE: Phosphatidylethanolamine; PG: Phosphatidylglycerol; and PI: Phosphatidylinositol.

In relative terms, CEs, TGs and PCs constitute over 65% of the lipid classes detectable in rabbit plaque. However, another relatively abundant lipid class detected in plaque, which is not appreciably detected in plasma, is the SMs [9]. As shown in Figure 3, SMs account for roughly 20% of plaque lipids. Within this lipid class, SM 16:0 is unequivocally the most abundant SM, most likely originating from PC 34:2 (Figure 2D, Table S4). SM 16:0, together with the next five most abundant SMs (SM 24:0, SM 24:1, SM 16:1, SM 22:0 and an SM 24:1 variant) account for about 85% of detectable plaque SMs (Figure 2D, Table S4).

## 3. Discussion

Whole body lipid homeostasis is regulated through the exogenous uptake and the endogenous synthesis of fatty acids and cholesterol, as well as the trafficking of these lipids to the appropriate anatomical depots. The perturbation of any number of mechanisms in these pathways can lead to the development of dyslipidemia, thereby increasing the propensity for lipid delivery and retention within the artery wall. Although the macromolecular components and major neutral lipid species of atherosclerotic plaques have been extensively defined [3,4,11], the lipidome of atheromatas has not been reported. Our goal was to produce a detailed framework of the lipids that comprise the atherosclerotic lesion of a widely used pre-clinical model of plaque progression, with particular attention to the most abundant lipid classes found in plasma.

Lipoprotein lipase (LPL) is the primary enzyme responsible for the liberation of FAs from lipoprotein particles, whereas hepatic lipase (HL) is the key enzyme responsible for the rapid removal of the resultant lipoprotein remnant particles from the circulation [12]. It is well established that rabbits are HL deficient, which causes extreme cholesterol accumulation within chylomicron remnant and β very low-density lipoprotein (β-VLDL) particles as a result of lowered metabolic clearance rate, when rabbits are challenged with a HC diet [12]. Although the balance between LPL and HL activities regulates lipoprotein clearance, the lipid composition of lipoprotein particles is primarily owing to the lecithin:cholesterol acyltransferase (LCAT) and acyl-coenzyme A:cholesterol acyltransferase (ACAT) enzymes, which are responsible for the conversion of FC to CE. Lipoprotein-bound LCAT cleaves sn2 position FAs (preferentially PUFAs) of PCs and transfers them to the 3-β-hydroxyl group of FC, thereby generating CE [13]. ACATs use MUFAs as their preferential substrate in the addition of exogenous dietary FAs or endogenous FAs generated through DNL, to FC [14]. The fact that we found predominantly dietary FAs (namely SFAs and MUFAs) within CEs of rabbit atherosclerotic lesions, suggests that ACAT is the major enzyme for the biosynthesis of CEs deposited in plaque. Moreover, our findings are consistent with the verity that ACAT-derived CE is the major atherogenic lipid in plasma [15], whereas LCAT-derived CE is the major atheroprotective lipid in blood, as it is mainly carried within high-density lipoprotein (HDL) particles [13]. Interestingly, our previous study demonstrated a higher dietary influence on plasma CE than on the plaque CE we observed in this study [9]. This discrepancy could potentially be due to macrophage metabolism of CE-derived FAs to other FA subspecies within the site of the lesion [16], warranting further lipidomic analyses of models of atherogenesis.

The data presented in this study are consistent with two recent reports which have demonstrated that TGs constitute a relatively abundant lipid species within rabbit atherosclerotic plaques [6,7]. Although challenging these animals with a HC diet does not elevate their plasma TG levels [9], TG accumulation within the atherosclerotic lesion has long been suggested to occur as a consequence of chylomicron and β-VLDL remnant retention in the artery wall [17], as well as DNL in the vascular intima from precursors such as acetate, glucose, and long-chain free fatty acids [18,19,20]. Our results are perhaps more consistent with the former, as we found that over 80% of the FAs within plaque TGs were nonessential FAs. Thus, our data suggest that lipoprotein-derived TGs are the major driver of plaque TG deposition. While* in situ* lipogenesis may certainly contribute at least part of the remaining portion of lesion TG, further studies would be required to delineate the exact contributions of lipoprotein* versus* lipogenic TG sources.

Phospholipids are major precursors to a host of signaling lipids, including eicosanoids and SMs [21], both of which are known to be involved in proinflammatory responses in atherogenesis [22,23]. In turn, their FA composition within atherosclerotic plaques is of particular interest. According to the Lands cycle, the ratio of saturated to unsaturated FAs within PCs in particular, is tightly regulated, resulting in a saturated FA occupying the sn1 position and an unsaturated FA occupying the sn2 position [24,25]. The result, at least in plasma, is an approximately equal distribution of FAs from nonessential and ω-6/ω-3 pathways in PCs [9]. In contrast, the distribution of plaque PCs is heavily slated towards the nonessential FA class, suggesting that FAs from other lipid classes within the lesion may influence plaque PC composition. For example, the generation of sphingomyelin results from the transfer of phosphorylcholine from PC to ceramide, which liberates diacylglycerols as a byproduct [26]. Given our finding that sphingomyelins and diacylglycerols are relatively abundant lipid classes within the lesion (Figure 3), the interplay between PCs, SMs and DGs may have influenced the observed distribution of FAs in plaque PCs. Furthermore, the relatively equal abundance of PCs and SMs (~20% of plaque, each) further supports the relationship between these lipid classes within the plaque. Therefore, the complex interactions between lipid classes in plaque warrants further study, as biomarkers of atherosclerosis progression, and perhaps changes in lipid profiles observed during plaque regression, may point towards biomarkers which can be used to validate therapeutics that modulate the disease.

## 4. Materials and Methods

### 4.1. Animals and Diets

Male New Zealand White rabbits (*n* = 20) were obtained from Covance (Princeton, NJ, USA) and were individually housed in a temperature- and humidity-controlled environment with a 12 h light/dark cycle. Animals were fed purified chow (Purina Mills LabDiet 5326, LLC, St. Louis, MO, USA) with 0.5% added cholesterol ad libitum for 12 weeks and had free access to water. At the end of the study, rabbits were euthanized with 1 mL/kg of Beuthanasia-D solution (Merck, Whitehouse Station, NJ, USA) via the marginal ear vein. Plasma lipid levels were determined on EDTA-plasma as described previously [9]. Full-length aortae were excised from each animal beginning with the aortic root through the aortic arch and the descending aorta to the iliac bifurcation. Aortae were dissected free of fat and connective tissue, flash-frozen in foil packets using liquid nitrogen and stored at −80 °C until future analyses. All animal experiments, procedures and protocols, were reviewed and approved by the Merck Research Laboratories’ Institutional Animals Care and Use Committee.

### 4.2. Lipid Extraction and Reagents

All organic solvents used were of chromatographic grade or better. Leucine enkephalin (Sigma Aldrich, St. Louis, MO, USA) solution was used for lock mass correction. Lipids were extracted from full-length aortae as per a modified Bligh and Dyer method as described previously [27,28]. During the homogenization and extraction process, samples were treated with anti-oxidants (0.5 mg of 2,6-di-tert-butyl-4-methylphenol). Extracts were evaporated to dryness and re-constituted in isopropanol/acetonitrile/water (65%:30%:5% (*v*/*v*/*v*)).

### 4.3. Analytical Instrumentation for Relative Quantitation of Lipid Fractions

Rabbit plaque lipid extracts were analyzed using an Acquity UPLC system coupled to a Synapt G2-HDMS mass spectrometer system (Waters Corp., Milford, MA, USA). For analyses, 5 µL of extract was injected. Chromatographic separation was achieved using a mobile phase A solution of 60% acetonitrile/40% water with 10 mM ammonium formate solution, and a mobile phase B solution of 90% isopropanol/10% acetonitrile with 10 mM ammonium formate. An HSS T3 C18 reverse-phase column was utilized (1.7 µm, 2.1 mm × 100 mm; Waters Corp.) coupled to the following chromatographic conditions: A linear gradient from 40% B to 100% B in 0 to 10 min followed by a 2 min hold at 100% B. The mass spectral data was acquired in electrospray positive and negative ion modes using nitrogen as the desolvation gas with a flow rate of 700 L/h. The source and desolvation temperatures were maintained at 120 and 450 °C, respectively. The capillary voltage, source cone voltage, and extraction cone voltage were maintained at +/−3 kV, +/−30 V, and 4 V, respectively. Mass spectra were acquired over the *m*/*z* range of 50 to 1200 at a resolution of 25,000 FWHM (full width half height mass resolution).

## 5. Conclusions

The observations presented in this study demonstrate the ability to obtain specific diet-induced lipid phenotypes from directly within the atherosclerotic plaque. However, these measurements do not provide any specific insights into the pathophysiology of the disease. For example, it is possible that *in situ* DNL and/or the metabolism of CE-, TG-, PC- and SM-derived FAs gives rise to signaling lipids such as oxylipin eicosanoids; known mediators of onset and progression of atherosclerosis. Although this example is speculative based on the present study, the data presented here provide the first complete characterization of the atherosclerotic plaque lipidome of a widely used *in vivo* model of the disease. This detailed framework of the lipids within the vessel wall provides a powerful lipid profile to enable future studies to expand on the lipidomic characterization of atherosclerotic lesions.

## Supplementary Materials

Supplementary tables can be found at http://www.mdpi.com/1422-0067/15/12/23283/s1.
